# Three-dimensional assessment of image distortion induced by active cardiac implants in 3.0T CMR

**DOI:** 10.1038/s41598-024-61283-0

**Published:** 2024-05-15

**Authors:** Theresa Reiter, Ingo Weiss, Oliver M. Weber, Wolfgang R. Bauer

**Affiliations:** 1 Department of Internal Medicine I, Cardiology, University Hospital Wuerzburg, Oberduerbacher Strasse 6a, 97080 Wuerzburg, Germany; 2 German Heart Center Munich, Electrophysiology, Munich, Germany; 3 BIOTRONIK SE & Co. KG, Berlin, Germany; 4 Philips GmbH, Hamburg, Germany

**Keywords:** Artifacts, Susceptibility, CMR, Active implants, Distortion, Imaging techniques, Cardiac device therapy

## Abstract

CMR at 3.0T in the presence of active cardiac implants remains a challenge due to susceptibility artifacts. Beyond a signal void that cancels image information, magnetic field inhomogeneities may cause distorted appearances of anatomical structures. Understanding influencing factors and the extent of distortion are a first step towards optimizing the image quality of CMR with active implants at 3.0T. All measurements were obtained at a clinical 3.0T scanner. An in-house designed phantom with a 3D cartesian grid of water filled spheres was used to analyze the distortion caused by four representative active cardiac devices (cardiac loop recorder, pacemaker, 2 ICDs). For imaging a gradient echo (3D-TFE) sequence and a turbo spin echo (2D-TSE) sequence were used. The work defines metrics to quantify the different features of distortion such as changes in size, location and signal intensity. It introduces a specialized segmentation technique based on a reaction–diffusion-equation. The distortion features are dependent on the amount of magnetic material in the active implants and showed a significant increase when measured with the 3D TFE compared to the 2D TSE. This work presents a quantitative approach for the evaluation of image distortion at 3.0T caused by active cardiac implants and serves as foundation for both further optimization of sequences and devices but also for planning of imaging procedures.

## Introduction

Cardiac MRI (CMR) is a cornerstone of non-invasive diagnostic methods in cardiology, allowing an unmatched overview of cardiac structure and function^1^. Likewise, active cardiac devices are an irreplaceable treatment option for patients at risk for a sudden cardiac death^2^.

However, the interferences of the MRI scanner’s magnetic fields with the components of pacemakers (PM) and implantable cardioverter defibrillators (ICDs) used to cause malfunctioning and damaging of these devices, consequently leading to exclusion of patients with active implants from CMR examinations^3–7^.

Recently established technical advances implemented in so-called MRI conditional devices have overcome these safety related issues in a well-defined setting, and devices for both 1.5 and 3.0T are nowadays available. Guidelines regarding procedure planning and patient monitoring enable access to MRI examinations for patients with these implants^2,8^. However, CMR in the presence of active cardiac implants remains a challenge for the clinical routine. In close proximity to these implants, extensive susceptibility artifacts occur that may overlap with the cardiac region, frustrating a diagnostic interpretation of the obtained images^9,10^.

For CMR examinations at 1.5T, protocol modifications have minimized this overlap of artifacts with the region of interest, not only for native CINE imaging, but also for contrast enhanced imaging techniques. The most recent clinical data demonstrate the value of late enhancement imaging employing a wideband inversion pulse for the treatment and prognosis of patients with ICDs, resulting in the modification of the diagnosis for 36% and changes in the treatment regime for 28% of the examined patients^11^.

The transfer of these modified imaging techniques to 3.0T scanners with their benefit of a higher signal-to noise ratio has yet to be established. The higher field strength allows for fast image acquisition and high image resolution^12^. However, the extent of the artifacts are dependent on and increase with the static magnetic field strength of the MRI scanner, consequently preventing in many patients the application of CMR at 3.0 T in the presence of active implants^9,13–16^.

The artifacts are the result of B0 field inhomogeneities induced by differences in susceptibility between materials and are characterized by different qualities. The dependence of the signal void, i.e. the complete loss of imaging information near the implant, was recently addressed by us^17^. However, image alterations extend beyond the region of (near-) complete signal cancellation and can manifest in a number of ways such as signal intensity variation or geometric distortion. The quantification of these effects requires a more subtle analysis. Homogeneous phantoms similar to the proposed setup by the ASTM standard are intended to quantify the device induced signal void^18^. However, due to the homogenous background, a three-dimensional quantification of distortion is not adequately possible. Even though there are phantom setups proposed, currently, there is no established standard for such a phantom^19–21^.

The evaluation and quantification of all artifact qualities can be helpful in optimizing CMR imaging and give insights regarding diagnostic interpretability of anatomical structures in the vicinity of different implant types. Here we present a quantitative approach that is based on a 3D cartesian grid geometry serving as reference and a thereon applied diffusion–reaction algorithm, for segmentation and alignment of image artifacts.

## Methods

### Phantom

**Figure 1:**
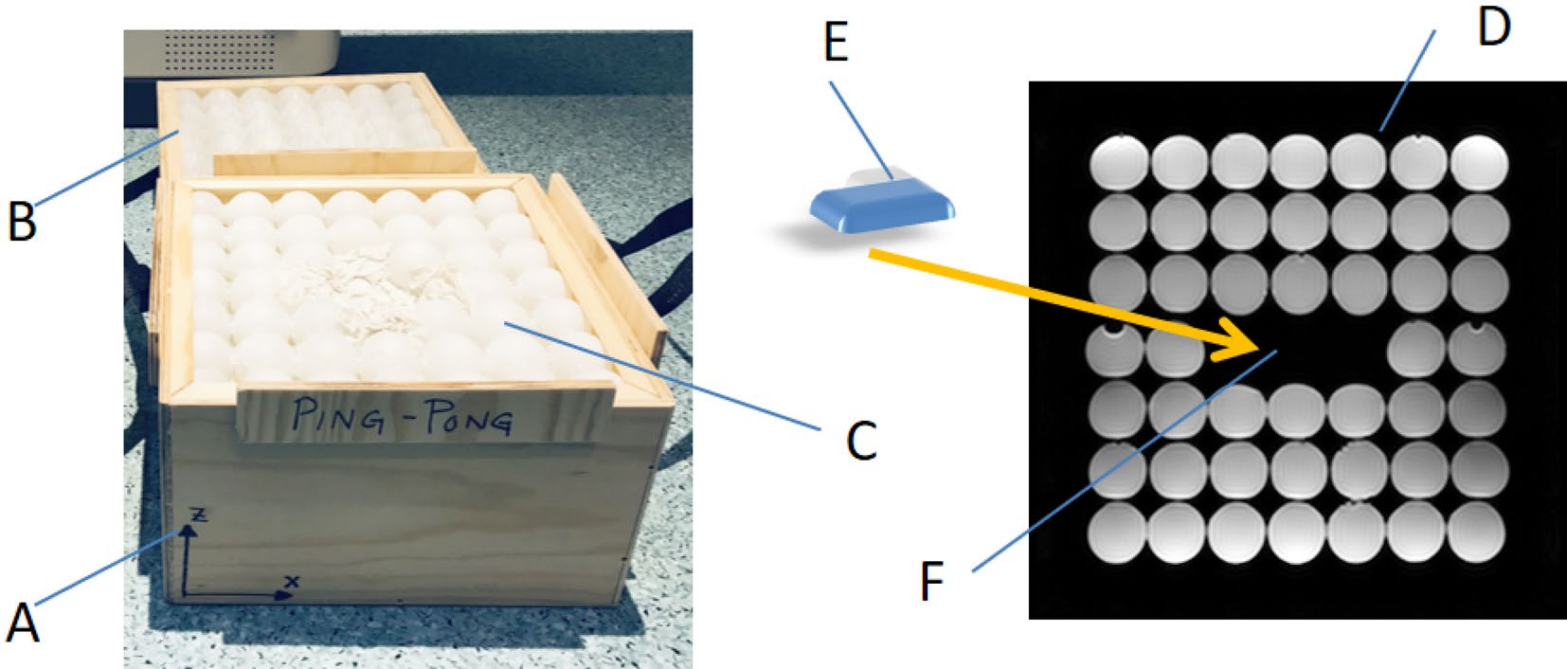
Phantom. The cubic phantom consists of a bottom part A and a top part B allowing positioning of the implant E in the preformed space F in the middle slice (D, yellow arrow). The spheres C are filled with plain water and are glued together for better mechanical stability. For the measurements, the top part is mounted on the bottom part. The phantom is marked with a coordinate system allowing a defined orientation. All measurements were performed in the orientation: X: left- right, Y: foot-head, Z: bottom up.

In order to detect the three-dimensional image distortion caused by active cardiac implants a cubic phantom with a grid-like structure was designed (Fig. 1). The outer dimensions of 28 × 28 × 28 cm were limited by the 60 cm bore diameter of the scanner. A cartesian grid formed by 7 × 7 × 7 spheres with a nominal diameter of 40 mm (40.17 ± 0.03 mm) for each sphere and a distance between the centers of two spheres of 40 mm was mounted into the phantom. By leaving out five spheres in the middle section, a central space for positioning of the devices was provided. The spheres (ping pong balls) consist of a thin plastic shell and were filled with plain water. The diameter of each sphere is significantly less than the wavelength at 3.0T, and thus excludes resonance interference effects.

### Devices

Four active cardiac implants were selected that represent the application spectrum of device therapy and differing amount of magnetic material (Biotronik. Germany). The representative implants included a cardiac loop recorder (Dev 1: Biomonitor 2), a pacemaker (Dev 2: Enticos 4 DR) and two ICDs (Dev 3: Ilesto 7 HF-T, Dev 4: Acticor 7 HF-T QP). Of the two ICDs, the former was optimized for reduced susceptibility artifacts^17^.

Measurements were performed without leads attached because their susceptibility artifacts are considered negligible.

### Measurement setup

All measurements were performed on a clinical 3.0T MRI scanner (AchievaDS, Philips Healthcare, Best, The Netherlands). Commercial anterior and posterior body surface coils were used for signal reception (dStream Whole-Body, Philips).

The imaging protocol has been discussed elsewhere^17^. In short, the protocol included a generic gradient echo and a generic spin echo sequence. Both sequences were slightly adopted from the ASTM publication F2119-07^18^. The main imaging parameters are listed in Table 1.

**Table 1:** Main imaging parameters.

	Grad echo	Spin echo
Dimensions	3D	2D MS
Image type	FFE	TSE (5 echoes)
TR (ms)	20	17,560
TE (ms)	3.2	27
Echo spacing (ms)	n/a	9.1
Flip angle (deg)	20	90
Flow comp	Yes	yes
Field of view (mm)	352	352
Acq. matrix	176	176
Nr of slices	176	176
Spat. resol. (acq) (mm^3^)	2 × 2 × 2	2 × 2 × 2
BW (Hz/pix)	382.4	473.5
Acq. dur. (mm:ss)	10:24	21:04
Spat. resol. (recon) (mm^3^)	1 × 1 × 1	1 × 1 × 2
FoV dir	RL	RL
Fat shift direction	P	P
Orientation	tra	tra
Max B1_rms (uT)	0.89	1.62
SAR level (W/kg)	< 0.4 W/kg	< 1.3
Db/dt (T/s)	50.4	38.9

Prior to all measurements with implants, reference scans without positioned implants were obtained. Due to nonlinearities of the gradient system, spheres especially at the borders of the FOV appear slightly distorted even in the absence of a cardiac implant. In order to quantify the distortion provoked solely by the presence of an active cardiac implant, these measurements are evaluated relatively to the reference scans. All spheres of the phantom were included in the image analysis process.

For the artifact measurements, the implant was positioned at the isocenter of the scanner with the device header in cranial-lateral orientation. The long axis of the cardiac loop recorder was positioned in left–right orientation on the implant holder.

### Image processing

The image data were stored in DICOM format as volume data or as a set of slices. All subsequent processing was performed with custom software scripted in MATLAB (The Math Works, Inc., MATLAB, version 2020a (Natick, MA: The Math Works, Inc., 2020)^22^). First the data were converted to 3D matrices and resampled to 2 × 2 × 2 mm voxel resolution. These steps were necessary to represent all data on the same grid structure for comparability. An exposure correction was performed by histogram stretching to correct for differences in image brightness and to be able to apply the same gray threshold for all data.

For segmentation of each sphere, an algorithm based on the region growing principle was introduced. Details of the implemented reaction diffusion equation are presented in the supplemental material.

#### Metrics

The metrics introduced for quantitative evaluation of the geometric distortions refer to the following properties which might be affected by the artifacts:

- Position of the spheres
- Shape of the spheres
- Gray values

From a different point of view the metrics can be classified as:

Comparative metrics (referring to changes of ball parameters due to the presence of the implant relative to the reference measurement of the phantom). Representative metrics are the differences between the center locations (dCL), the volumes (dV), between the out-of-round measures (dORM) and between the gray values (dGV).

- Non-comparative metrics (referring to absolute values of parameters that describe the spheres geometry). Representative metrics are the volume (V) and the out-of-round measure (ORM).

The ball center is defined by the gravity center coordinates of the voxels assigned to the respective sphere. To quantify by how much spheres appear displaced due to distortions the distance dCL between homologous spheres is calculated as a comparative metric. The ball volume V is calculated as the sum of the ball voxel volumes. The growth or shrinking of structures is expressed as volume differences of homologous spheres (reference vs. phantom with implant):

$${\text{dV}}=\frac{{{\text{V}}}_{{\text{d}}}-{{\text{V}}}_{{\text{r}}}}{{{\text{V}}}_{{\text{r}}}},$$

where ${{\text{V}}}_{{\text{d}}}$ is the volume of the distorted ball and ${{\text{V}}}_{{\text{r}}}$ the volume of its homologous reference representation. The out-of-round measure is defined:

$${\text{ORM}}=\frac{{{\text{R}}}_{{\text{M}}}}{{{\text{R}}}_{{\text{m}}}}-1,$$

where ${{\text{R}}}_{{\text{M}}}$ is the average of the upper 5th percentile of the radii. The radii are calculated as the distance from the ball center to the voxels on its surface the ball. The surface voxels are identified by a gradient operation. Similarly ${{\text{R}}}_{{\text{m}}}$ is calculated for the lower 5th percentile of the radii. Percentile and average calculation was performed to eliminate outliers due to potentially rough surfaces of the segmented spheres. In case of a perfect sphere ORM = 0, while OMR = 1 indicates that the longest dimension of the distorted ball is twice as large as the shortest one. The comparative metric

$${\text{dORM}}={{\text{ORM}}}_{{\text{d}}}-{{\text{ORM}}}_{{\text{r}}},$$

expresses changes of the ball roundness. Relative changes of gray values (averaged over the ball volume) are calculated for homologous spheres:

$${\text{dGV}}=\frac{{{\text{GV}}}_{{\text{d}}}-{{\text{GV}}}_{{\text{r}}}}{{{\text{GV}}}_{{\text{r}}}}.$$

In the immediate vicinity of the implant that equals the center of the phantom, the extent of image distortion is the highest, and decreases with distance. The critical radius (Rcr) defines the region where these implant induced image distortions are above a predefined threshold value. The threshold value is twice the uncertainty level thus ensuring a reasonable SNR. For a conservative approach, the critical radius, the nominal radius of the phantom’s ball spheres (20 mm) is added to the threshold value.

### Uncertainty assessment

The uncertainty assessment is based on four scans of the phantom without positioned implant. The first scan served as reference and the results of the three following reproductions were compared to the first one. The volumes of the reconstructed spheres were averaged, and the wall thickness of the spheres was estimated to be 0.2 mm. Even though the spheres’ diameter has a very small tolerance (Ping Pong balls, 40.17 ± 0.03 mm), the reconstructed volumes vary with the position within the phantom and the chosen imaging sequence.

The equivalent radii from the minimum and maximum reconstructed spherical volumes were calculated and the difference was used as surrogate of uncertainty in space dimensions.

## Results

The analysis of the geometrical distortions makes use of the changes in position and shape of the reconstructed spheres in the phantom.

**Figure 2:**
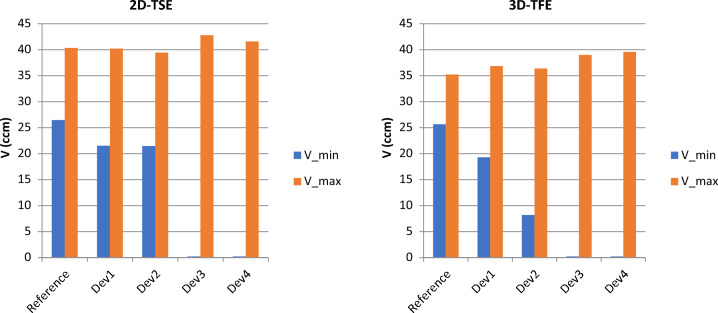
Impact of the implant type and the scanning sequence on the volumes of the reconstructed spheres. V_min and V_max of the reference scan shows the systemic distortion at the borders of the field of view. For Dev1 and Dev2, none of the spheres completely disappears from the field of view. whereas in case of Dev3 and Dev 4, some spheres appear completely suppressed.

The presence of an active cardiac implant induces both, a reducing and an increasing effect on the volumes of the spheres in the vicinity of the implant. The reducing effect (Vmin) can occur to an extent that the affected spheres almost disappear. This effect is more pronounced for implants with higher amount of magnetic material. The increasing effect (Vmax) equally occurs in the vicinity of the implant. However, the absolute values differ only little from the reference scans because the enlarged spheres are partially concealed by the implant’s signal void and only a fraction of the real absolute volume is reconstructed. The maximum values range from 39.4 and 42.8 cm^3^ in the 2D-TSE scans and from 36.8 to 39.5 cm^3^ in the 3D-TFE scans (Fig. 2).

**Figure 3:**
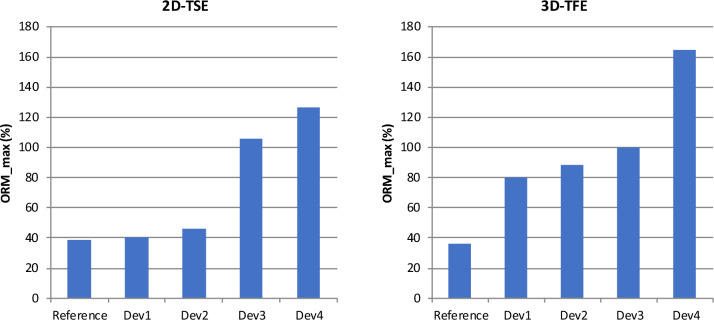
Impact of the implant type and the scanning sequence on out-of-round measure (ORM) of the reconstructed spheres. For the 2D-TSE, Dev1 and Dev2 show only very little ORM, whereas Dev3 and Dev4 show a significant increase. For the 3D-TFE, all devices show significantly more ORM than the reference scan.

The out-of-round measure (ORM) increases with the amount of magnetic material (Fig. 3). In case of the 2D-TSE sequence, the ORM shows little difference from the reference for Dev 1 and Dev 2; however, the value more than doubles in the presence of Dev 3 and Dev 4. The 3D-TFE reacts much more sensitive with regard to this parameter. Even in the presence of Dev 1 and Dev 2, the ORM increases significantly. In the presence of Dev3, the ORM increase further but remains markedly below the values for Dev 4.

**Figure 4:**
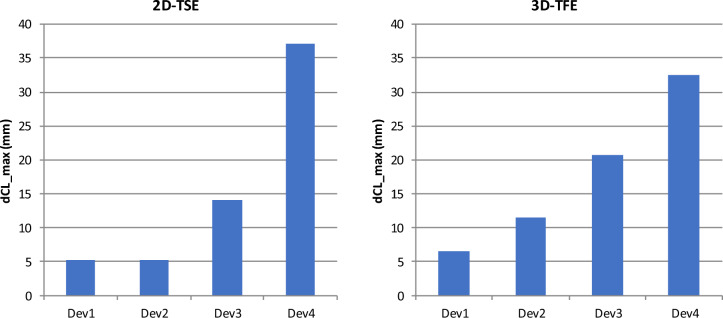
Impact of the implant type and the scanning sequence on the maximum shift of the reconstructed sphere centers. For the 2D-TSE, Dev1 and Dev2 induce a maximum displacement of 5 mm, whereas Dev4 shows a displacement of 37 mm. For the 3D-TFE, the displacement caused by Dev2 more than doubles.

Figure 4 displays the maximum shift of the sphere centers as a consequence of the image distortion. While both sequences produce lagers shifts for implants comprising more magnetic material, the effect is more pronounced with the 2D-TSE. The data also show relevant differences between Dev 3 and Dev 4.

**Figure 5:**
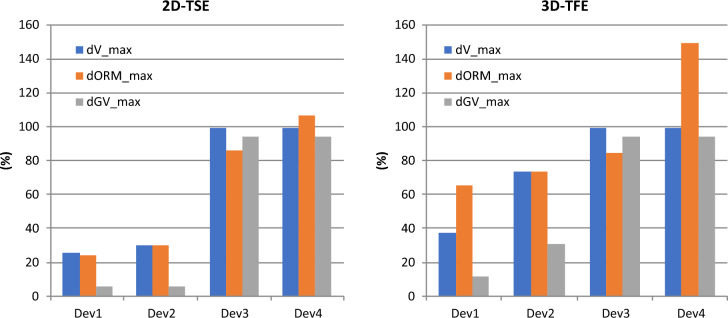
Impact of the implant type and the scanning sequence on the changes relative to the reference regarding the entities volume, out-of-round measure and gray values of the reconstructed spheres.

The metrics dV_max, dORM_max and dGV_max depend on the device type as well. The 2D-TSE again results to be more sensitive to differences in the amount of magnetic material, however, the artifacts are generally more pronounced with the 3D-TFE (see Fig. 5).

**Figure 6:**
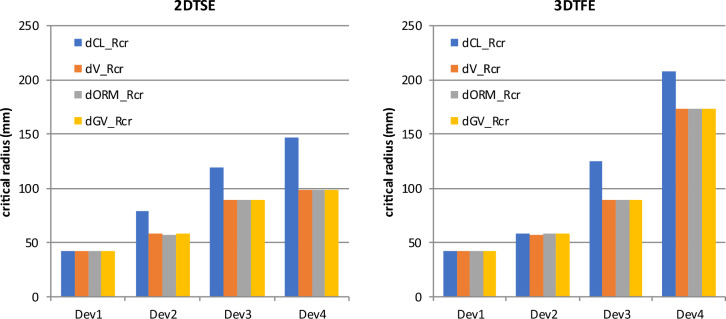
Impact of the implant type and the scanning sequence on the critical radius up to which a measurable effect can be demonstrated.

The critical radii are display in Fig. 6. They also depend on the amount of magnetic material and are more pronounced for the 3DTFE sequence.

**Figure 7:**
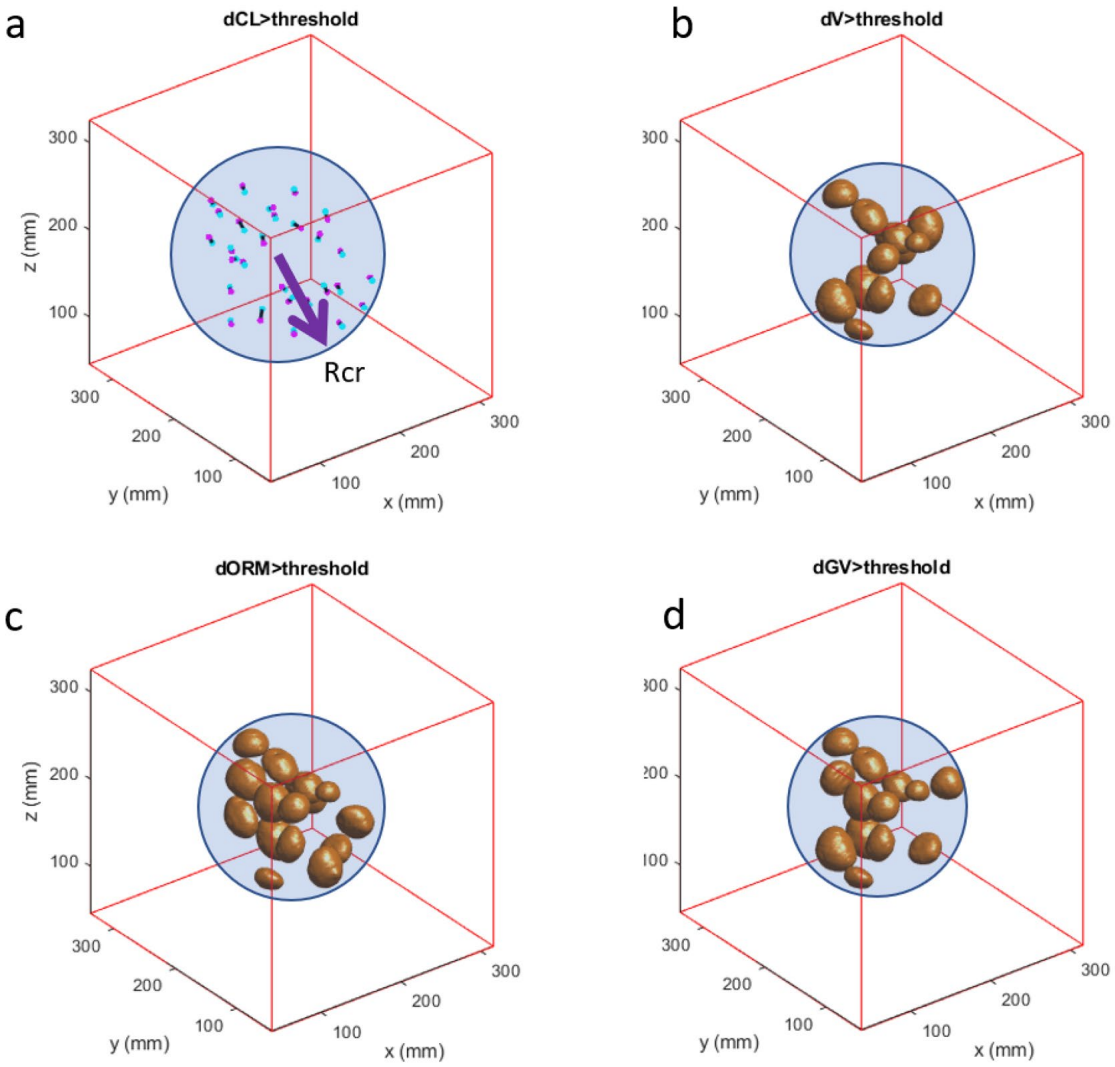
Exemplary result for Dev3 scanned with 2D-TSE showing the reconstructed spheres inside the critical radius Rcr. (**a**) sphere centers shifted by more than the threshold value from light blue dot (reference) to magenta dot as indicated by the black lines, (**b**) spheres affected by change in volume lager than a threshold value, (**c**) spheres affected by an ORM change lager than a threshold value, (**d**) spheres affected by gray value changes lager than a threshold value. The spheres outside of the critical radius are not shown.

**Figure 8:**
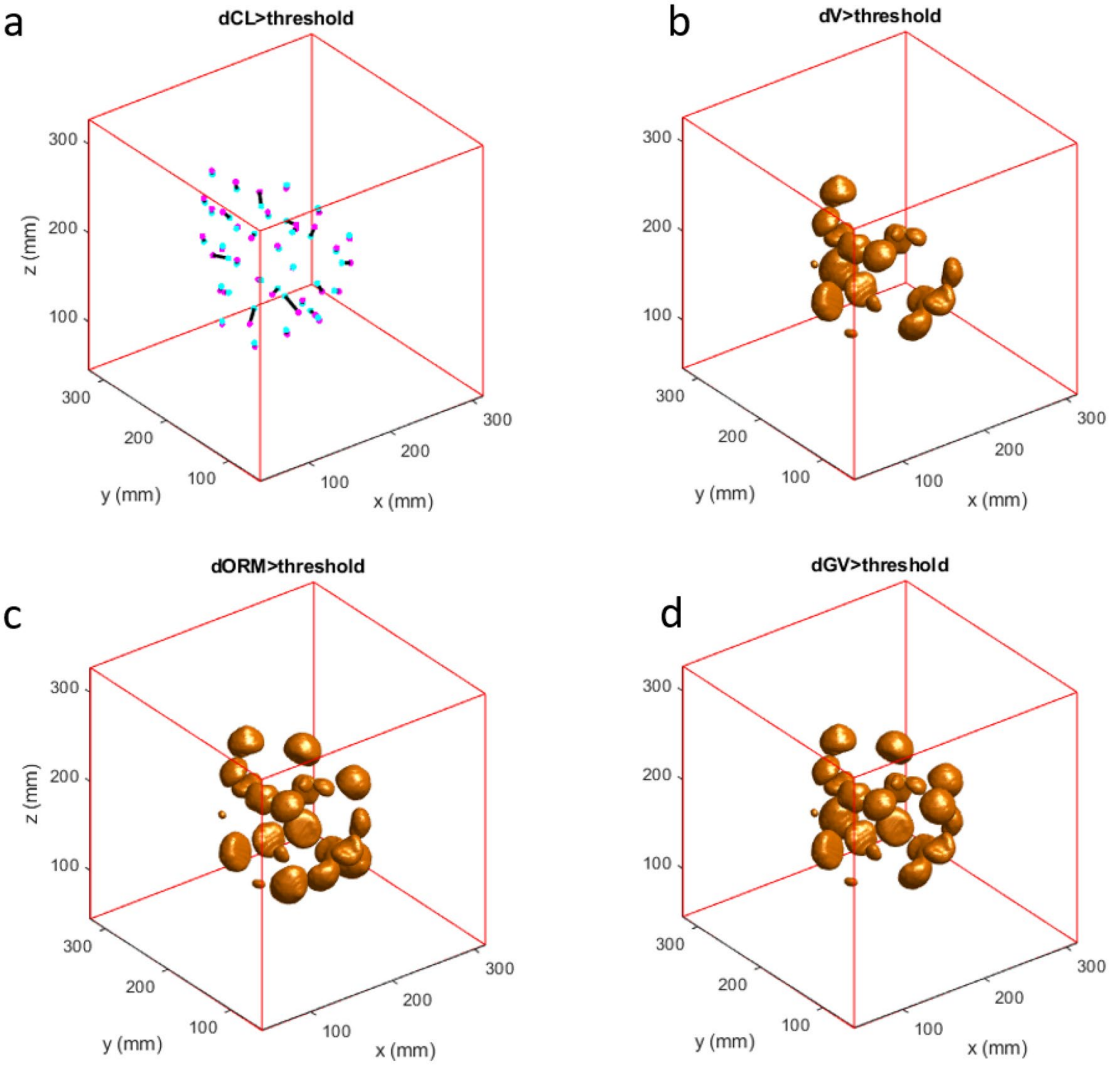
Exemplary result for Dev3 scanned with 3D-TFE showing the reconstructed spheres inside the critical radius. (**a**) sphere centers shifted by more than the threshold value from light blue dot (reference) to magenta dot as indicated by the black lines, (**b**) spheres affected by change in volume lager than a threshold value, (**c**) spheres affected by an ORM change lager than a threshold value, (**d**) spheres affected by gray value changes lager than a threshold value. The spheres outside of the critical radius are not shown.

Finally, Figs. 7 and 8 show some examples of ball displacements and morphological deformations due to impact of Dev3. The figures also show the special distribution of distortion effects in the vicinity of the implant. The distortion effects are not evenly distributed around the implant, and do not follow geometrical principles. Differences between the image sequence types are recognizable. Appendix Fig. 4 shows representative MRI scans. For the values, see Appendix Table 1.

### Uncertainty assessment

Although the used Ping-Pong balls had very tight tolerances (40.17 ± 0.03 mm, nominal water volume 32.9 ccm) the volumes of the reconstructed spheres in the reference scans varied between 24.6 and 40.3 ccm (standard deviation 2.57 ccm; 36.1–42.6 mm equivalent sphere diameter) for the 2DTSE and 25.3–35.3 ccm (standard deviation 1.45 ccm; 36.4–40.7 mm equivalent sphere diameter) for the 3DTFE. The average volumes are 31.7 ccm for the 2DTSE and 31.1 ccm for the 3DTFE, respectively, and are a quite good estimate for the actually expected water volume of 32.9 ccm. Calculating the equivalent radii from the minimum and maximum reconstructed volumes the uncertainty in space dimensions results to be almost 3.5 mm. Therefore, the threshold for the critical radius concerning placement errors is set to 7 mm.

The uncertainties of the comparative metrics dV_max, dORM_max and dGV_max resulted to coincide for both imaging sequences having the values 12%, 9%, and 2% respectively. The thresholds for the corresponding critical radii are therefore 24%, 18%, and 4% respectively.

## Discussion

Even in the absence of active cardiac implants, CMR at 3.0T can be a challenge compared to scans at 1.5T. At the higher field strength, increased differences in the susceptibility of neighboring tissues cause larger artifacts, and this effect is markedly more pronounced in the presence of active implants^12^. Qualitative analyses demonstrate the different qualities of these induced susceptibility artifacts such as signal void and image distortion. Especially the latter is a challenge because it potentially introduces false image information due to magnifying or shrinking effects as well as deviations in the signal intensity^23,24^. At 1.5T, a phantom study that focused on the effects of orthopedic implants on MRI images calculated an impressive theoretical signal distortion of up to 28 mm depending on material, distance to the implant and chosen sequence, but also more subtle distortions of less than 2 mm were measured^20,21,25^. In case of CMR, a distortion of 2 mm can arguably be neglected. However, a distortion of up to 28 mm equals the mean diameter of the thoracic and abdominal aorta, and thus very well might influence the image interpretation regarding additional non cardiac structures or the heart itself^26^).

For the quantitative evaluation of distortion, different types of phantoms have been proposed, using e.g. a grid of small spherical fiducial with a diameter of 6 mm or a custom designed grid system with cylindrical structures^20,21^. However, these concepts, albeit highlighting important features of distortion, do not fully represent the complex features of distortion. Small spheres are limited with regards to changes in shape and displacement, whereas larger spheres not only allow detection of this effect but also a truly three- dimensional quantification and also depict changes in the optical impression represented by the grey values. The design of our phantom allows the analysis of very different aspects of distortion during one measurement thus recreating the different aspects of distortion seen (or overlooked) in in vivo measurements. The image processing balances setting and sequence dependent differences in brightness, and by using the reaction diffusion based segmentation algorithm it is robust against distortion caused shunts between sphere volumes. Furthermore, it is flexible regarding shape and number of segmentation volumes.

Similar to the results obtained in a previous work the artifact burden increased with the device's ferromagnetic components^17^. However, for each implant, the distortion was significantly higher when the 3D–TFE instead of the 2D-TSE sequence was used. Even in devices with as little amount of ferromagnetic material as an event recorder, the changes in the grey values increase more than 6 times when compared to the results obtained with the 2D-TSE.

The data show the uneven distribution of distortion effects within the critical radius. The image data beyond this distance from the implant shows no relevant distortion, however, within this radius, displacement, changes of the grey value and minimizing or maximizing effects can be detected. These do not follow a linear relationship to the increasing distance and are not equally distributed within the critical volume. Similar to the prior analysis of the signal void caused by the active cardiac device, the comparison of two ICD models show marked differences in the amount of artifacts despite being the same device type^17^. Dev 3 induces far less artifact burden with the 3D-TFE sequence than Dev 4, equaling in its artifact burden smaller devices such as pacemakers. Among the analyzed qualities, the shifting of the center location is the most prominent effect with a critical radius of 12.5 cm for Dev 3 and 20.7 cm for Dev 4 when measured with the 3D-TFE sequence. Consequently, for Dev4, the anatomical region that is affected by the distortion effect, is markedly larger than for Dev3. This finding points towards another important aspect when establishing CMR imaging at 3.0T for patients with active cardiac implants. Besides the influence of the sequences, the devices themselves greatly influence the artifact burden, and as demonstrated with the two different models of ICDs, the design of the devices might offer another target for optimization.

Our data show that the reliability of image information is not solely based on the increasing distance from the implant. Even though the image appearance might be satisfactory, at least within the critical radius, image interpretation should be performed with caution.

In conclusion the current work presents a reliable approach to segmentation and quantification of image distortion in the setting of 3.0T CMR in the presence of active cardiac implants. The definition of critical radii for each artifact quality allows an estimate for the expected clinical imaging result and offers a foundation for further imaging optimization.

### Limitations

The current study focuses on four implants that cover the available cardiac device therapy. However, as has been shown, there are differences between different devices of the same manufacturer, and more are to be expected when other manufacturer’s devices are exposed to the CMR.

### Supplementary Information

Supplementary Information.

## Data Availability

The datasets used and analysed during the current study are available from the corresponding author on reasonable request.
