# Supplementary material for: Three-dimensional assessment of image distortion induced by active cardiac implants in 3.0T CMR
Source: Sci Rep. 2024 May 15;14:11130. doi: 10.1038/s41598-024-61283-0 (PMC11096309; doi:10.1038/s41598-024-61283-0)
Supplement: Supplementary file 1 — Supplementary Information. [file 41598_2024_61283_MOESM1_ESM.docx]

Appendix

#### Data segmentation

To assess the geometric distortions a phantom is used having water filled spheres arranged as a cartesian grid. This regular grid makes distortions easily detectable. To determine metrics quantifying the distortions the spheres must be segmented first to have access to the voxels of each sphere individually. The segmentation method is designed for high computational performance and is based on the region growing principle implemented by solving a reaction-diffusion equation. Starting from a seed a reaction-diffusion process spreads through the volume until the growth is limited by material properties. The seed is first located in the nominal sphere center position. The reaction-diffusion equation is formulated as:

| $\frac{\partial u}{\partial t}=D\cdot\Delta u+k\cdot u\cdot\left( 1-u \right)$ | (eq.1) |
| --- | --- |

where the state variable u is the reactant. The diffusion constant D of the computation medium is set accordingly to the gray values. For the dark background outside the spheres with gray values below a threshold (see slice view in Fig 1 of the main manuscript). The D-value is set to zero to prevent diffusion outside the spheres. The reaction term is chosen of the type

| $\rho=k\cdot u\cdot\left( 1-u \right)$ | (eq.2) |
| --- | --- |

with k=0.5, to assure limited growth in magnitude. This is required for subsequent threshold operation to be image content independent. Eq. 1 is solved numerically by discretizing it on the Cartesian voxel grid. In this case the Laplace operator can be computed by convolution with a 3x3x3 kernel kD defined as

kD=zeros(3);

kD(1,2,2)=1;

kD(2,1,2)=1;

kD(2,2,1)=1;

kD(3,2,2)=1;

kD(2,3,2)=1;

kD(2,2,3)=1;

kD(2,2,2)=-6;

using MATLAB notation. The integration in time is performed using the Euler method. A reasonable time step was determined to guarantee numerical stability.

Due to limited image resolution, image imperfections and implant induced distortions the spheres may appear interconnected. Therefore, the reaction starting in one of the spheres may spread to neighboring spheres. However, the leakage spot is tiny and there additionally the diffusion is reduced (darker gray values), hence, the region growth speed will go down. This mechanism helps defining a stop criterion for the diffusion and prevents that adjacent spheres glue together. Appendix figure 1 shows how the reactant u spreads into neighbor spheres. To prevent this undesired effect the growth is observed over time by evaluating:

| $U\left( t \right)=\iiint_{Ω} u\left( t \right)dv$ | (eq.3) |
| --- | --- |

where $Ω$ is the computation domain.


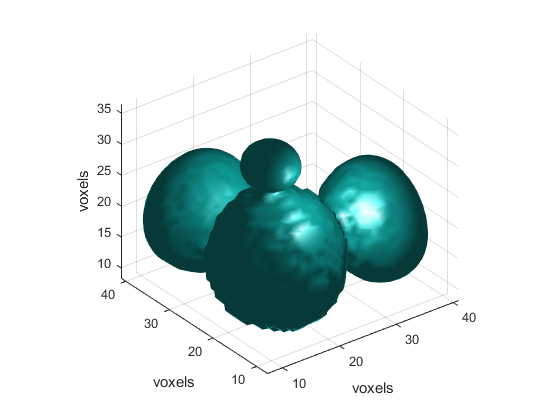


Appendix-Figure 1: Example where the reactant u spreads into neighboring spheres

The reaction-diffusion process is stopped when

| $U_{2}=\frac{d^{2}U}{dt^{2}}<0$ and $U_{3}=\frac{d^{3}U}{dt^{3}}>0$ | (eq.4) |
| --- | --- |

for the first time. Appendix figure 2 helps understanding the stop criterion which is fulfilled when the first slow-down occurs.

Appendix- figure 2: Growth over time of the reactants volume integral U for the example in Figure 1 together with the second U2 and third U3 derivative

Finally, each sphere is identified base on the volume filled with the reactant at the timestep when the reaction diffusion process was stopped according to the described criterion. Appendix Figure 3 exemplarily shows two layers of segmented spheres of a reference scan.


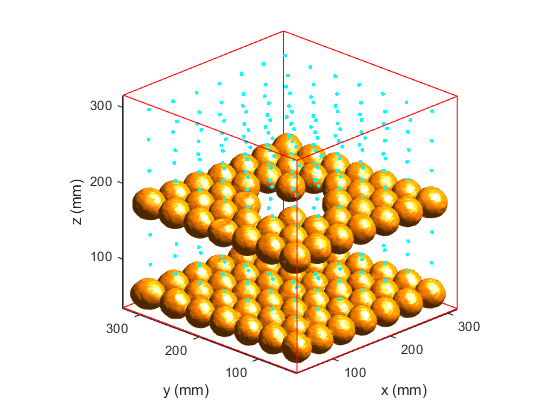


Appendix figure 3: Example of a reference scan with the sphere phantom showing two layers of segmented spheres. In the middle layer 5 spheres are missing to allow the placement of the implant. The light blue dots indicate the centers of the other segmented spheres not displayed in the figure.


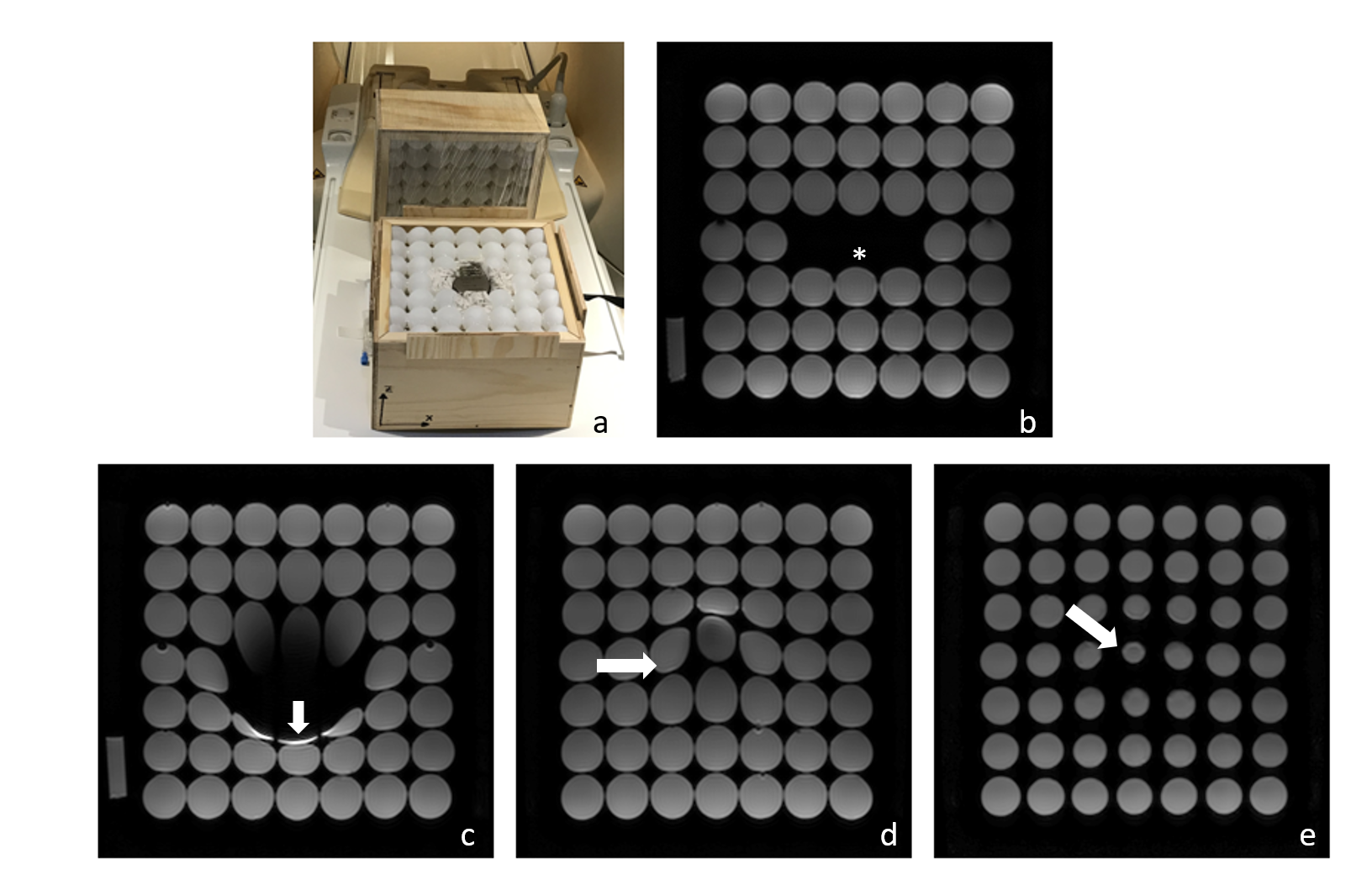


Appendix figure 4: Phantom( a) and MRI scans without (b, asterisk marks the space reserved for the implant) and with Dev 3 (c- d). c-d shows different slices in the vicinity of the implant. c) marks changes in the signal intensity. d) shows displacement and e) shows minimizing effects.

| Parameter | Dev1 | Dev2 | Dev3 | Dev4 |
| --- | --- | --- | --- | --- |
|  |  |  |  |  |
| dV_max [%] | 25,83 | 30,09 | 99,26 | 99,31 |
| dV_min[%] | 0,09 | 0,02 | 0 | 0 |
| dV_sd[%] | 3,35 | 3,92 | 9,46 | 14,1 |
| dV_mean[%] | 4,63 | 3,45 | 5 | 8,42 |
|  |  |  |  |  |
| dORM_max[%] | 24,39 | 29,7 | 85,67 | 106,28 |
| dORM_min[%] | 0,0011 | 0,0114 | 0,0029 | 0,0165 |
| dORM_sd[%] | 1,74 | 3,02 | 8,99 | 13,45 |
| dORM_mean[%] | 1,08 | 1,66 | 3,44 | 6 |
|  |  |  |  |  |
| dGV_max[%] | 5,7 | 5,56 | 93,8 | 93,11 |
| dGV_min[%] | 0,0009 | 0,0006 | 0,0002 | 0 |
| dGV_sd[%] | 0,64 | 0,7 | 5,52 | 8,52 |
| dGV_mean[%] | 0,9 | 0,68 | 1,3 | 2,46 |
|  |  |  |  |  |
| dV_Rcr [mm] | 41,65 | 57,83 | 89,52 | 98,01 |
| dORM_Rcr [mm] | 41,65 | 57,31 | 89,52 | 98,39 |
| dGV_Rcr [mm] | 41,65 | 57,83 | 89,52 | 98,18 |
|  |  |  |  |  |

Appendix Tab 1: Values of dORM, dV and dGV . Given are maximum, minimum, standard deviation and mean values, as well as the critical radii in mm.
